# Oxygen Defect-Induced Metastability in Oxide Semiconductors Probed by Gate Pulse Spectroscopy

**DOI:** 10.1038/srep14902

**Published:** 2015-10-08

**Authors:** Sungsik Lee, Arokia Nathan, Sanghun Jeon, John Robertson

**Affiliations:** 1 Electrical Engineering Division, Department of Engineering, University of Cambridge, Cambridge CB2 1PZ, United Kingdom; 2 Department of Applied Physics, Korea University, 2511 Sejong-ro, Sejong-si, 339-700, Republic of Korea

## Abstract

We investigate instability mechanisms in amorphous In-Ga-Zn-O transistors based on bias and illumination stress-recovery experiments coupled with analysis using stretched exponentials and inverse Laplace transform to retrieve the distribution of activation energies associated with metastable oxygen defects. Results show that the recovery process after illumination stress is persistently slow by virtue of defect states with a broad range, 0.85 eV to 1.38 eV, suggesting the presence of ionized oxygen vacancies and interstitials. We also rule out charge trapping/detrapping events since this requires a much smaller activation energy ~0.53 eV, and which tends to be much quicker. These arguments are supported by measurements using a novel gate-pulse spectroscopy probing technique that reveals the post-stress ionized oxygen defect profile, including anti-bonding states within the conduction band.

Transparent conducting oxides are promising candidate as channel material for thin film transistors (TFTs) due to their high electron mobility and optical transparency^1^,^2^,^3^. However, this class of materials shows persistent photoconductivity (PPC) even under visible illumination despite its wide band-gap ~3 eV^4^,^5^. Interestingly, PPC can give rise to high optical detectivity in oxide semiconductor-based photodetectors^5^,^6^,^7^,^8^,^9^. Thus its imperative that we understand the physical origin of PPC.

In order to explain the underlying mechanisms of PPC, there are two possible models. One relates to charge trapping (CT) and the other to oxygen defect ionization (ODI) (see Fig. 1(a)). With the former^6^,^9^, optically generated holes can be trapped at the gate insulator and its interface, and in defects in the oxide semiconductor layer (e.g., localized traps in an amorphous oxide structure, and grain boundary defects in nano-crystalline oxides). The trapped holes can induce electrons in the channel, yielding a negative threshold (V_T_) shift in the drain current (I_DS_) vs. gate-voltage (V_GS_) characteristics in an oxide TFT^5^,^6^,^7^,^8^,^9^,^10^. However, the PPC can also be explained with the ODI model. Here, a negative V_T_ shift implies more electrons (e^−^) are created thereby increasing Fermi level (E_F_). These additional electrons (2e^−^) can originate from the ODI process in the form of metastable oxygen defects, such as oxygen vacancy ionization (V_O_^0^ → V_O_^2+^ + 2e^−^) and oxygen interstitial ionization (I_O_^2−^ → I_O_^0^ + 2e^−^)^5^,^7^,^8^,^9^,^10^,^11^,^12^,^13^,^14^,^15^ (see Fig. 1(b)). These two ionization processes can be formulated commonly as D^0/2−^ → D^2+/0^ + 2e^−^. The ionized oxygen defects (D^2+/0^) remain ionized since they are at a higher energy level compared to their previous states (D^0/2−^), as illustrated in Fig. 1(b). This makes the shift in V_T_ persist, thus slowing down recovery and forming the physical basis of PPC. Thus the origin of illumination-induced instability (i.e. negative V_T_ shift) can appear to be somewhat controversial between the CT and ODI mechanisms.

This work attempts to clarify this controversy through a series of experiments and analyses using amorphous In-Ga-Zn-O (a-IGZO) TFTs. Firstly, the recovery behavior after illumination stress is measured independently and compared with the case after negative bias stress (NBS) in the dark. The latter is intended to promote the hole trapping^16^,^17^,^18^,^19^,^20^,^21^. Here, the bias and time of NBS are tuned to −20 V and 30 sec, respectively, to yield an equivalent value of negative V_T_ shift when subject to illumination stress for 30 sec duration. It is found that the recovery behavior after illumination stress is very slow (i.e. persistent) whereas NBS leads to a fast recovery. This demonstrates that the instability with illumination stress cannot be dominated with the hole trapping.

Secondly, we studied activation energy distributions for stress and recovery with illumination stress and NBS, respectively. With the former, the TFT’s gate electrode was kept floating to minimize the vertical E-field which can be a source of hole trapping. To get the activation energy profile, we used a stretched exponential function to model stress and recovery characteristics as a function of time, and its inverse Laplace transform with the Arrhenius relation. As a key observation, the recovery process after illumination stress appears to happen through two different defect states. One defect state provides a much higher activation energy level compared to the other, which has a similar energy distribution with the recovery behaviour after NBS. These results indicate that the state located at the higher energy level is the main origin of the slow recovery and PPC. This further suggests that the recover process after illumination stress takes place through more deeply bound states, which can be explained with the ionized oxygen defect states.

We introduce a new probing technique based on gate-pulse spectroscopy (GPS) to capture the profile of these metastable oxygen defects, e.g. ionized oxygen defect states, as a function of energy. Here, a gate-pulse train with an incremental pulse height from 1 V to 13 V is applied for gradual recovery of V_T_. The amount of recovered V_T_ can be correlated with the concentration of the ionized oxygen defects. Although the extent of recovered V_T_ can also stem from electron trapping during the GPS procedure, we can subtract this using the values of V_T_ shift obtained from an equivalent GPS procedure applied to a non-illuminated sample. Based on this, we find that the profile of the ionized oxygen defect states is distributed in the energy range from −1.25 eV to +0.07 eV. In particular, the states within the conduction band at 0 to +0.07 eV can be viewed as anti-bonding states (σ*) between the lattice oxygen and excess oxygen atoms (interstitials).

Finally, we argue that the proposed analysis and spectroscopy technique are useful to identify optical instability mechanisms in other oxide semiconductors such as Zn-O, Ga-O, and In-Zn-O.

## Results and Discussion

### A. Instability Observations

**Figure 2:**
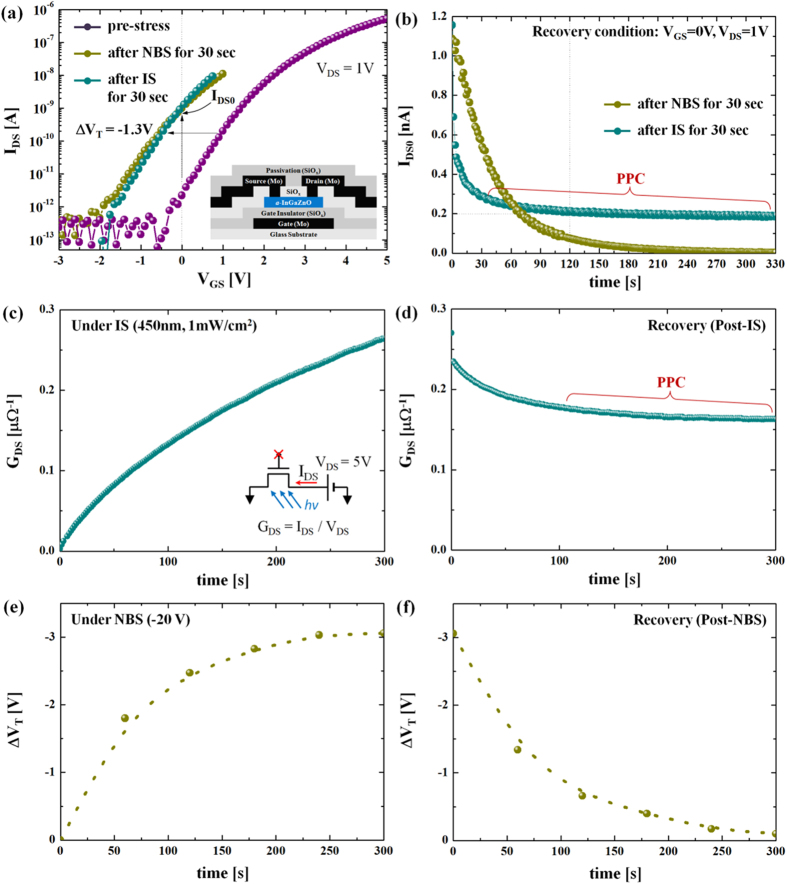
(**a**) Transfer characteristics at V_DS_ = 1 V for the cases after NBS (at V_G_ = −20 V) and illumination stress for 30 sec in comparison with the pre-stressed a-IGZO TFTs (Inset: Schematic cross-sectional view of the examined TFTs). The TFTs examined have channel length (L) = 4μm and width (W) = 100 μm with 100 nm thick SiO_2_ as the gate insulator. Both NBS and illumination stress lead to a negative ΔV_T_ of −1.3 V, and I_DS_ at V_GS_ = 0 V (I_DS0_) is increased as an outcome of the negative V_T_ shift. (**b**) Recovery behavior of I_DS0_ after illumination stress and NBS, respectively. (**c**) Measured conductance (G_DS_) as a function of time under illumination stress (photo-excitation) without gate probing as shown in the inset. (**d**) Measured G_DS_ as a function of time time after illumination stress (recovery). (**e**) Measured ΔV_T_ as a function of time under NBS (excitation). (**f**) Measured ΔV_T_ as a function of time after NBS (recovery). Here, ΔV_T_ values are retrieved from the I_DS_-V_GS_ characteristics (at V_DS_ = 1 V) measured with NBS at every 60 sec. After 300 sec of NBS, ΔV_T_ values are retrieved from the I_DS_-V_GS_ characteristics (at V_DS_ = 1 V) measured without NBS at every 60 sec.

Figure 2(a) shows the measured drain current (I_DS_) as a function of gate voltage (V_GS_) at a drain voltage (V_DS_) of 1 V for two different cases, one is after NBS for 30 sec and the other after illumination stress for 30 sec, in comparison with the pre-stress case. Here, the level of NBS is limited to −20 V to get the same threshold voltage shift (ΔV_T_ ~ −1.3 V) as with illumination stress at ~0.5 mW/cm^2^ optical power at 450 nm wavelength. At the same time, the corresponding drain current at V_GS_ = 0 V, denoted as I_DS0_ (used as a reference to ΔV_T_), is increased up to 1 nA for both cases, as seen in Fig. 2(a). This is also seen in Fig. 2(b) at t = 0 sec in which both stresses are removed and recovery processes start with the condition of V_GS_ = 0 V and V_DS_ = 1 V. We see that the recovery after NBS is almost complete while the recovery after illumination stress shows a persistency (i.e. PPC) and almost saturating after 120 sec, although the initial decay is fast. This initial fast decay may be due to detrapping of holes that may have been trapped in the gate insulator or interface during illumination. It can be argued that the illumination-induced V_T_ shift and PPC, as evidenced by the observed recovery, is not totally arising from the hole trapping.

However, the fast and complete post-NBS recovery, as seen in Fig. 2(b,f)^16^, can be explained with the hole detrapping. To quantify this, the detrapping time (τ_d_) for each hole can be estimated as^17^,


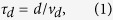


where *d* is the characteristic penetration depth at which traps are filled with holes, and ν_d_ the drift velocity for holes. Here, v_d_ can be defined as v_d_ = μ_h_·E_eff_ = 3 × 10^4^ cm/s, where the hole mobility μ_h_ ≈ 0.1 cm^2^/V·s^16^, and the transverse built-in electric field post NBS E_eff_ ≈ |ΔV_T_(0)|/t_ox_ = 3 × 10^7^ V/m for ΔV_T_(0) ≈ 3 V and thickness of gate insulator t_ox_ ≈ 100 nm. In Equation (1), d is defined as follows^17^, d = 1.15|ΔV_T_(0)|/(K_d_r_d_). Here, the wave function decay constant K_d_ ~ 10^10^/m, and the decay rate r_d_ ≈ 1.7 V/dec calculated as *d*|ΔV_T_(t)|/*d*log(t) on the decay plot seen in Fig. 2(f), yielding d ≈ 0.2 nm. Finally, Eq.(1) gives τ_d_ ≈ 6.7 × 10^−12^ sec, implying a fast recovery. In addition, it has been demonstrated that this fast recovery process of trapped holes is fast enough high frame rate active matrix organic displays^18^.

For the illumination stress experiments shown in Fig. 2(a,b), a vertical electric field (E-field) along the channel depth is induced by virtue of an applied positive gate bias. This E-field can give rise to charge trapping. To minimize the effect of the gate field and to extract a pure channel layer contribution to illumination-induced instability, the transient conductance (G_DS_) with the gate floating is measured with a fixed V_DS_ = 5 V (see the inset of Fig. 2(c)). As seen in Fig. 2(c), the G_DS_ under illumination stress is increased with time, suggesting an increase of the channel layer conductivity (σ_ch_) since G_DS_ ≈ σ_ch_t_ch_W/L, where t_ch_ is the channel thickness. After illumination stress for 300 sec, the recovery of G_DS_ is also monitored, as seen in Fig. 2(d). Similar to Fig. 2(b), G_DS_ is not completely recovered and shows PPC behavior. In contrast, the trend of ΔV_T_ in post-NBS shows almost complete recovery with a behavior that is symmetric with that during NBS—see Fig. 2(e,f). To quantify the observed stress and recovery behaviour, a stretched exponential analysis is employed^19^,^20^. This is discussed in the following section.

### B. Stretched Exponential Analysis

**Figure 3:**
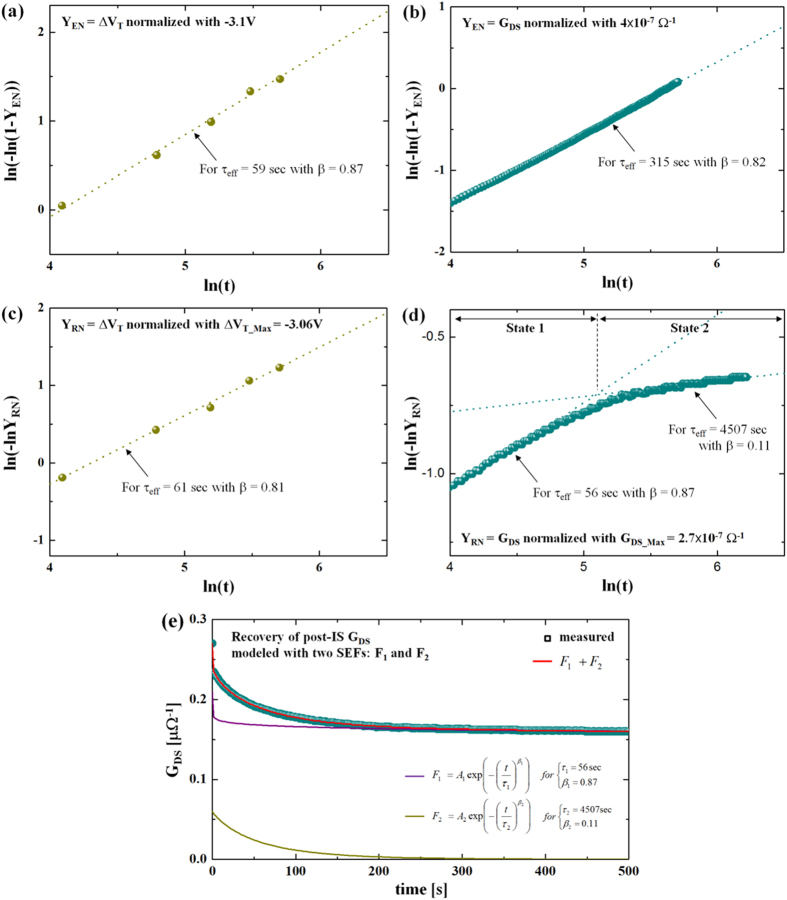
Excitation behavior modeled with SEF for (**a**) NBS and (**b**) illumination stress, respectively. Recovery behaviour modeled with SEF for (**c**) NBS and (**d**) illumination stress, respectively. (**e**) Recovery behaviour as a function of time for post illumination stress G_DS_ modeled with two SEFs. Here, A_1_ and A_2_ are coefficients, and their sum equals to G_DS-Max_.

To model the transient stress and recovery characteristics, we use a stretched exponential function (SEF) of time (F(t)) as follows^19^,^20^,


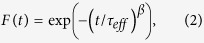


where τ_eff_ is an effective time constant and β a stretched exponent which is a real number bigger than zero. Note that normalized stress and recovery behavior can be represented as 1-F(t) and F(t), respectively, and the common term to explain each transient curvature is F(t) which mainly determines τ_eff_ and β. Based on Equation (2), the stress and recovery curves for both illumination stress and NBS can be modeled, yielding values of τ_eff_ and β for each case, as seen in Fig. 3(a–d) (see also the section S1 in the Supporting information). As expected, the post-NBS case shows good agreement with a single SEF, implying presence of a single mechanism, i.e. hole detrapping. In contrast, the post illumination stress case shows a piece-wise linear characteristic. Thus this recovery behavior should be modeled with two different SEFs, as shown in Fig. 3(d,e), implying presence of two independent mechanisms during post illumination stress recovery. The first SEF has τ_eff_ = 56 sec and β = 0.87, which are quite comparable with the post-NBS case, suggesting presence of a similar mechanism during the post illumination stress recovery process. At the same time, the second SEF yields a value of τ_eff_ = 4507  sec, which is larger compared to any other case, and β = 0.11. In particular, this large value of τ_eff_ implies the need for a much longer time period to finish recovery. This is reminiscent of persistent photoconductivity and an important bearing on the role of oxygen defects. The extracted values of τ_eff_ and β for each case are summarized in Table 1. With these values, we can now deduce an activation energy distribution.

### C. Activation Energy Distribution

To get an activation energy distribution function, as the first step, we need to find the frequency domain function, f(S), from the inverse Laplace transform (ILT) of F(t)^21^ defined as follows,


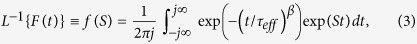


where S is a frequency. In order to convert this to an energy distribution function, the frequency parameter S needs to be replaced with activation energy (E_A_) using the Arrhenius relation,


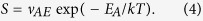


Here, ν_AE_ is an attempt-to-escape frequency and kT the thermal energy. Note that ν_AE_ is different from a lattice vibration frequency which is about 10^13^/sec^22^,^23^. Using a saddle point method along with Equation (4)^24^, the analytical solution of Equation (3) constitutes the activation energy distribution function, f(E_A_),


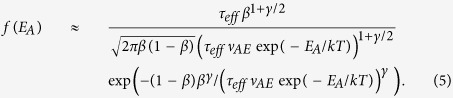


For the detailed derivation procedure, see the section S2 in the Supporting Information. In Equation (5), the main unknown is ν_AE_. To retrieve the value of ν_AE_, excitation state experiments under illumination stress are performed for different temperatures. This yields ν_AE_ = 10^7^/sec (see section S3 in the Supporting Information), suggesting that the examined device has ~10^7^/sec as an attempt-to-escape frequency, which is consistent with the value chosen in ref. 23.

**Figure 4:**
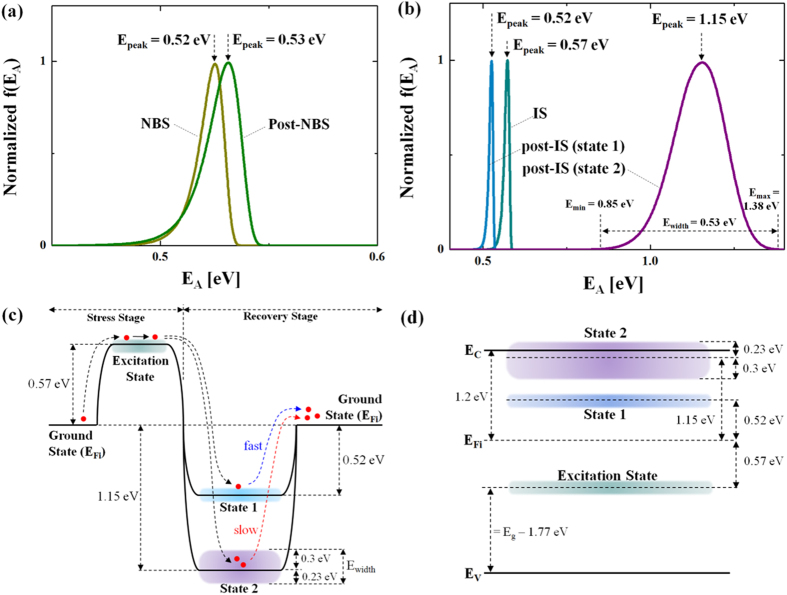
Normalized f(E_A_) as a function of activation energy (E_A_) for (**a**) NBS (excitation) and post-NBS (dark recovery), and (**b**) illumination stress (photo-excitation) and post illumination stress (dark recovery), respectively. (**c**) Activation energy diagram for the cases of illumination stress (photo-excitation) and post illumination stress (dark recovery). Here, the ground state level is defined as an intrinsic Fermi level (E_Fi_). (**d**) Deduced location of each state as a function of energy. In particular, State 2 has both states below and above the conduction band minima (E_C_).

With the extracted value of ν_AE_, we can plot the activation energy distribution function f(E_A_) for each of the instability cases using Equation (5) using the respective values of τ_eff_ and β (see Table 1). Figure 4(a) shows the normalized f(E_A_) for NBS (excitation) and post-NBS (dark recovery) cases, respectively. The peak energy level (E_peak_) under NBS is 0.52 eV, which is similar to that of the post-NBS, suggesting complete recovery in a similar time period. However, the cases of illumination stress (photo-excitation) and post illumination stress (dark recovery) are quite different from the NBS cases. As seen in Fig. 4(b), the post illumination stress has double peaks. In particular, it shows a much higher activation energy state (state 2) while state 1 is located near the excitation state. This implies that there are two different recovery processes. Since state 2 has a higher peak activation energy ~1.15 eV, it suggests that recovery is being processed through more deeply bound states^20^, and is consistent with the nature of the ionized metastable oxygen defects^4^,^5^,^6^,^7^,^8^,^9^,^10^,^11^,^12^. Here, we believe that the slow recovery after illumination stress, i.e. PPC, is due to the ionized oxygen defects, which needs a higher activation energy to be neutralized^11^,^12^. This is depicted in Fig. 4(c).

Here, the ground state level, defined as an intrinsic Fermi level (E_Fi_), is ~1.2 eV. The corresponding energy band diagram in Fig. 4(d) shows the location of each state as a function of energy. Here, the key observation is state 2 located on both sides of the conduction band minima (E_C_), in which states above E_C_ are supposed to be an anti-bonding state associated with oxygen interstitials^25^.

### D. Gate-Pulse Spectroscopy

**Figure 5:**
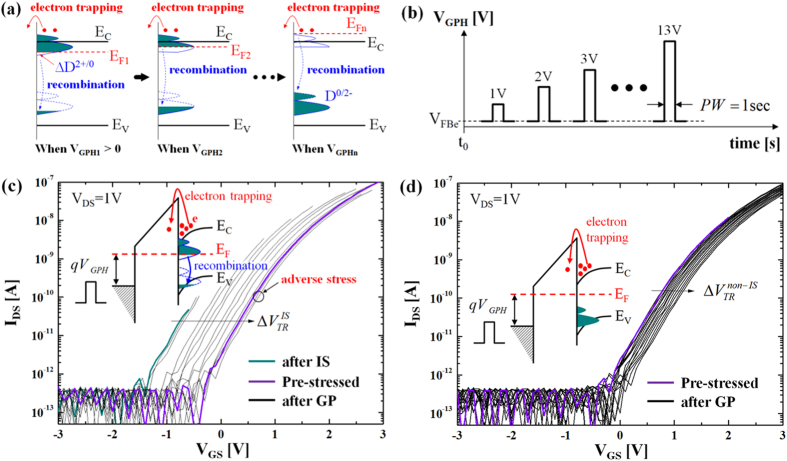
(**a**) Illustration to show the effects of the GP: electron trapping as well as recombination of the ionized oxygen defects. (**b**) Illustration of gate-pulse (GP) train with incremental pulse height (V_GPH_) from 1 V to 13 V (step = 1 V). (**c**) I_DS_-V_GS_ characteristics after applying each GP in comparison with virgin and the case after IS before applying GP, which measured after IS. (**d**) I_DS_-V_GS_ characteristics after each GP in comparison with virgin, which measured with non-IS samples.

For insight into the location of these defect states, we developed a new gate-pulse spectroscopy (GPS) technique which yields the DOS profile of the ionized metastable oxygen defects, e.g. ionized oxygen vacancies and interstitials. The GPS technique is applied after illumination stress. In particular, GPS allows us to compare the result shown in Fig. 4(d). Here, the basic idea is that the D^2+/0^ states become occupied when they are located below E_F_, hence enabling recombination with electrons, i.e. D^2+/0^ + 2e^−^ → D^0/2−^, and hence the recovery of V_T_. If we vary the gate pulse height (V_GPH_) in small increments, the D^2+/0^ states become gradually occupied, as depicted in Fig. 5(a). As an outcome after each gate pulse, V_T_ correspondingly recovers with a behavior that correlates with the density of ionized oxygen defects. Note that, besides oxygen defect recombination, it is also possible that there is electron trapping as an adverse effect while applying the GPS, as described in Fig. 5(a). After 60 sec illumination stress with 1 mW/cm^2^ optical source of 450 nm, the transfer characteristics show a negative V_T_ shift which is almost saturated yielding |ΔV_T_| = 1.2 V after 240 sec from the time at which illumination stress ceases (i.e. start of recovery). Subsequently, we apply a gate-pulse train with varying pulse heights from 1 V to 13 V, as shown in Fig. 5(b). Here, the gate-pulse height (V_GPH_) is set as V_GS_–V_FBe_ (where V_FBe_ is an effective flat-band voltage at which I_DS_ = 10^−13^ A). In our case, a complete recovery of V_T_ is achieved after V_GPH_ = 11 V, leading to a positive V_T_ shift after every stage of the gate-pulse train. However, it is found that transfer curves after gate pulsing with V_GPH_  ≥ 12 V are further shifted to a more positive direction compared to the pre-stressed case, as can be seen in Fig. 5(c), This implies presence of another mechanism stemming from the gate-pulse. Since the polarity of gate-pulse is positive, it may be due to the electron trapping (see Fig. 5(a)). This means the gate pulse-induced V_T_ recovery after illumination stress (ΔV_TR_^IS^) should contain some degree of electron trapping at every stage of the gate-pulse train, as depicted in the inset of Fig. 5(c). To estimate this, we perform gate pulsing on non-illuminated samples. As shown in Fig. 5(d), it is found that the transfer curve is shifted after each stage of the gate-pulse train. This is mainly due to the electron trapping. Here, we observe that the degree of V_T_ shift of the non-illuminated sample (denoted as ΔV_TR_^non-IS^) is much smaller than the illuminated counterpart (ΔV_TR_^IS^). Obviously the non-illuminated sample does not have ionized oxygen defects and any shift in V_T_ is solely due to electron trapping, as illustrated in the inset of Fig. 5(d). In other words, it can be argued that the gate pulsing on the sample after illumination stress gives rise to a bigger positive V_T_ shift arising from not only electron trapping but also from the recombination of photo-generated electrons with ionized oxygen defects.

**Figure 6:**
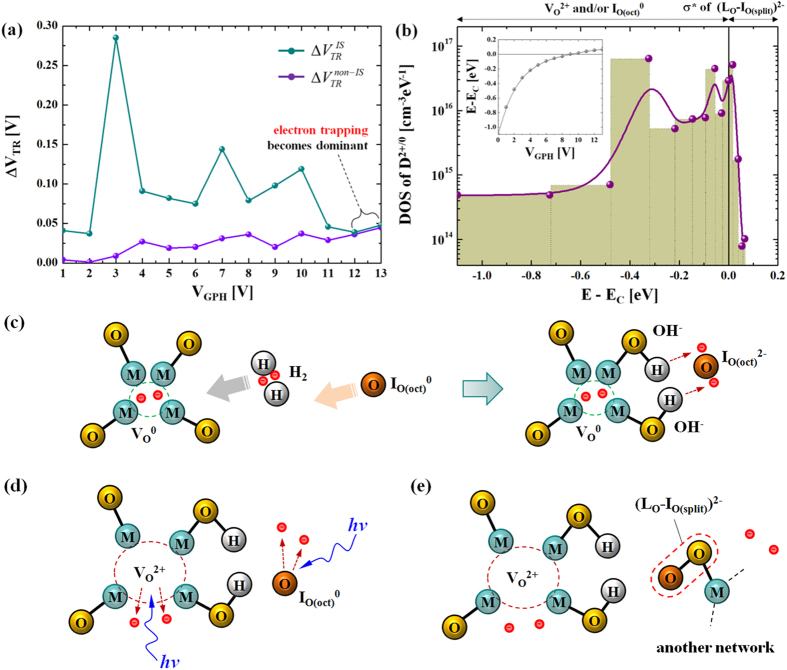
(**a**) V_T_ shift after illumination stress following application of a series of gate pulses (green line) in comparison with that of the non-illumination stress sample after applying the series of gate pulses (orange line). (**b**) Extracted DOS of the ionized oxygen defects (D^2+/0^) vs. energy from conduction band (E-E_C_). The inset: correspondence between E-E_C_ and V_GPH_. (**c**) Reaction between hydrogen and lattice oxygen. Here, electrons are donated from hydrogen, which are eventually used to occupy oxygen interstitials. (**d**) Illustration of the ionized oxygen interstitials (I_O(oct)_^0^) after illumination stress, as a possible source of the PPC. (**e**) Depiction of a further reaction between unoccupied oxygen interstitials (I_O(oct)_^0^) and lattice oxygen (L_O_^2−^), which yields (L_O_-I_O(split)_)^2−^ where is anti-bonding states located within the conduction band.

We have now extracted all the values of ΔV_TR_^IS^ and ΔV_TR_^non-IS^. This is summarized in Fig. 6(a). Here, the difference between ΔV_TR_^IS^ and ΔV_TR_^non-IS^ is the density of recombined D^2+/0^. As a key observation in Fig. 6(a), both values of ΔV_TR_^IS^ and ΔV_TR_^non-IS^ become similar when V_GPH_ > 11 V. This suggests that the dominant mechanism is now the electron trapping rather than oxygen defect filling, and retrieving the oxygen defect profile beyond V_GPH_ = 11 V may lead to ambiquities. From that information in Fig. 6(a), the density of the ΔD^2+/0^, i.e. n(ΔD^2+/0^), can be retrieved with the following equation 5,8, 9, 10,


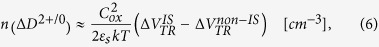


where ε_s_ is the permittivity of channel layer, C_ox_ the gate-insulator capacitance, and kT the thermal energy at ambient temperature. In addition, the distribution of the D^2+/0^ can be shown as a function of energy. For this, the V_GPH_ value needs to be translated to E-E_C_ using the surface potential, resulting in the following numerical relation^26^,^27^,


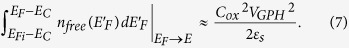


Here, the free carrier density n_free_ is numerically computed based on Fermi-Dirac statistics^28^. We assume that the ionized states below E_F_ are fully occupied and those above E_F_ are unoccupied. Solving Equation (7), the correspondence between E-E_C_ and V_GPH_ is given, as shown in the inset of Fig. 6(b). Based on this, we can now retrieve the density of states of the D^2+/0^ (i.e. N(D^2+/0^)) as a function of energy (E-E_C_), using the following equation,


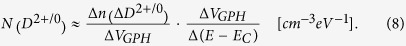


As shown in Fig. 6(b), it is found that the profile of the ionized oxygen defect states is distributed in the energy range from −1.25 eV to +0.07 eV (above the conduction band minima). This result is quite consistent with the results shown in Fig. 4(d). As for the nature of these ionized states (D^2+/0^) within the gap (−1.25 eV to 0 eV), we consider both unoccupied octahedral-like oxygen interstitials (I_O(oct)_^0^) and oxygen vacancies (V_O_^2+^) as labeled at the top in Fig. 6(b). These states are initially occupied by 2 electrons, thus I_O(oct)_^2−^, which can be donated by hydrogen (H_2_) forming OH^−^ with lattice oxygen (L_O_^2−^), as illustrated in Fig. 6(c)^29^. These interactions can be summarized as,


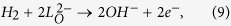



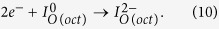


Before illumination, these octahedral oxygen interstitials are fully occupied, thus I_O(oct)_^0^. However, under illumination, electrons can be excited from the I_O(oct)_^2−^ states. This process yields neutral states (I_O(oct)_^0^)^29^,^30^,^31^. These unoccupied states can remain unoccupied even after removal of illumination, thus it can be a possible form of the D^2+/0^ (see Fig. 6(d)).

As another possible form of the D^2+/0^ related to I_O(oct)_^0^, anti-bonding states of (L_O_-I_O(split)_)^2-^ are considered. This split oxygen interstitials (I_O(split)_) are derived from the interaction between I_O(oct)_^0^ and lattice oxygen (L_O_^2−^)^25^. This forms anti-bonding states within the conduction band, providing two free electrons. This results in PPC. In our experimental results shown in Fig. 6(b), the states within conduction band from 0 to 0.07 eV can be thought of as these anti-bonding states (σ*), i.e. (L_O_-I_O(split)_)^2−^, between lattice oxygen (L_O_^2−^) and unoccupied octahedral oxygen interstitial (I_O(oct)_^0^)^25^ (see Fig. 6(e)).

## Conclusions

Experimental evidence suggests that the negative threshold voltage shift under illumination stress and its post-stress persistency is due to metastable oxygen defect (vacancy and/or interstitial) ionization as opposed to carrier trapping/detrapping. Indeed, the inverse Laplace transform of transient recovery behavior post illumination stress as compared to that obtained under illumination stress yields activation energy distributions for two independent types of defect states; one that resides at a significantly higher activation energy level compared to the other. Here, the higher activation energy states limit the recovery rate after illumination stress, thus giving rise to persistent photoconductivity. The arguments are further corroborated by findings from gate pulse spectroscopy, which yields the post stress oxygen defect profile and also presence of anti-bonding states between lattice and interstitial oxygen atoms in the conduction band.

## Additional Information

**How to cite this article**: Lee, S. *et al.* Oxygen Defect-Induced Metastability in Oxide Semiconductors Probed by Gate Pulse Spectroscopy. *Sci. Rep.* **5**, 14902; doi: 10.1038/srep14902 (2015).

## Supplementary Material

Supplementary Information

## Figures and Tables

**Figure 1:**
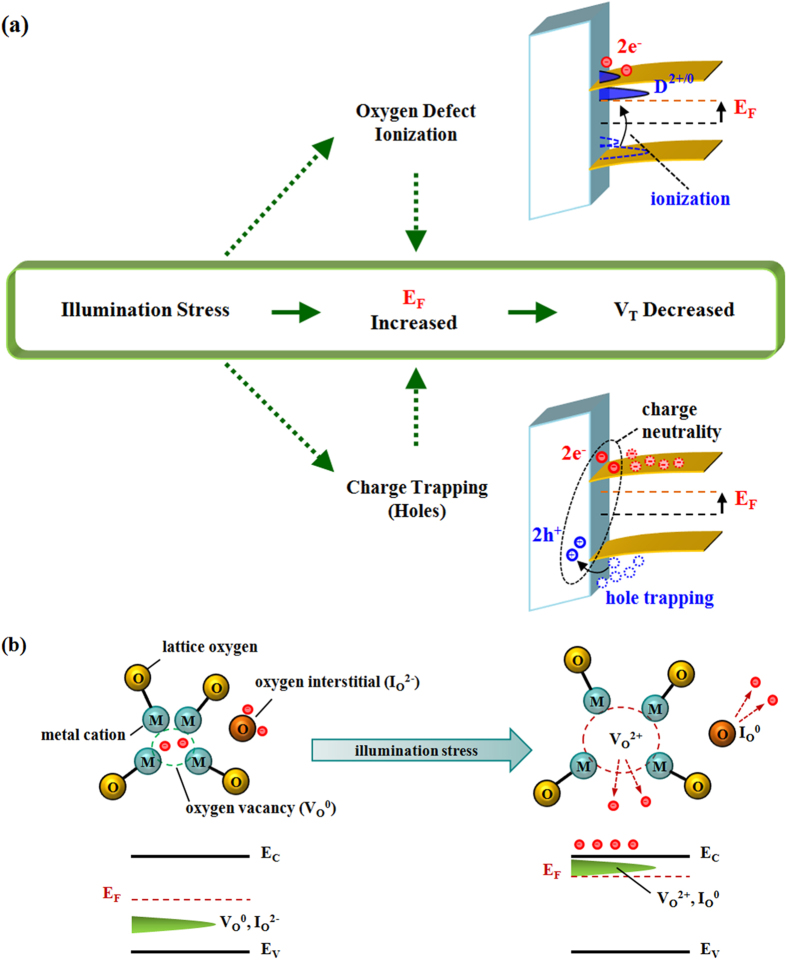
Illustration of (**a**) instability mechanisms and (**b**) oxygen defect ionization (ODI) to explain persistent conductivity associated with the increased energy level of the ionized oxygen defect states under illumination stress.

**Table 1:** Parameters Retrieved with a Stretched Exponential Function for NBS and Illumination Stress Cases.

StressType	Excitation		Recovery		
	τ_eff_	β		τ_eff_	β
NBS	59 sec	0.87		61 sec	0.81
LS	315 sec	0.82	F_1_	56 sec	0.87
			F_2_	4507 sec	0.11
