# Supplementary material for: Oxygen Defect-Induced Metastability in Oxide Semiconductors Probed by Gate Pulse Spectroscopy
Source: Sci Rep. 2015 Oct 8;5:14902. doi: 10.1038/srep14902 (PMC4597196; doi:10.1038/srep14902)
Supplement: Supplementary Information [file srep14902-s1.pdf]

Supplementary Information

Oxygen Defect-Induced Metastability in Oxide Semiconductors Probed by Gate Pulse Spectroscopy

Sungsik Lee[1], Arokia Nathan[1†], Sanghun Jeon[2], and John Robertson[1]

[1] Electrical Engineering Division, Department of Engineering, University of Cambridge, Cambridge CB2 1PZ, United Kingdom.
[2] Department of Applied Physics, Korea University, 2511 Sejong-ro, Sejong-si, 339-700, Republic of Korea.
(†: Corresponding Author)

List of Information:

S1. Stretched Exponential Analysis

S2. Activation Energy Distributions with ILT

S3. Determination of V_{AE}

S1. Stretched Exponential Analysis

To quantify and compare the stress and recovery plots for each IS and NBS case, a stretched exponential function (SEF) of time (F(t)) is employed,

$$F(t) = e^{-(t/\tau_{eff})^{\beta}}, \qquad \text{(S1-1)}$$

where τ_{eff} is an effective time constant and β a stretched exponent which is a real number bigger than zero. Note that normalized stress and recovery behavior can be represented as 1-F(t) and F(t), respectively, and the common term to explain each transient curvature is F(t). Based on Eq.(S1-1), the stress (i.e. excitation) and recovery curves for IS and NBS can be modeled, yielding values of τ_{eff} and β for each case.

The normalized excitation behaviour (Y_{EN}), as seen in Figs.2(c) & (e), can be modeled with |1-F(t)|. This means that the following expression can be made with the normalized excitation function (Y_{EN}),

$$Y_{EN} = 1 - F(t). \qquad \text{(S1-2)}$$

Eq.(S1-2) can be rewritten for graphical analysis to extract τ_{eff} and β, as follows,

$$\ln\left(-\ln(1-Y_{EN})\right) = \beta\left(\ln t - \ln \tau_{eff}\right). \qquad \text{(S1-3)}$$

Now, using Eq.(S1-3) and experimental date shown in Figs.2(c) & (e), excitation behaviours for NBS and IS are modeled as seen in Figs.S1-1(a) & (b), respectively. It provides a good agreement between experiments and SEF, yielding values of τ_{eff} and β for each case.

Similarly, the normalized recovery behaviour (Y_{RN}), as seen in Figs.2(d) & (f), can also be modeled with the following expression,

$$Y_{RN} = F(t), \qquad \text{(S1-4)}$$

$$\ln\left(-\ln Y_{RN}\right) = \beta\left(\ln t - \ln \tau_{eff}\right). \qquad \text{(S1-5)}$$

Figs.S1-1(c) & (d) show the modeled results with SEF for recovery behaviour of each post-NBS and post-IS. As normal, post-NBS case shows a good agreement with a single SEF as seen in Fig.S1-1(c). It implies that there is a single mechanism which is a hole detrapping. In contrast, the recovery bahaviour should be modeled with two different SEF, as shown in Fig.S1-1(d). This implies that there are two independent mechanisms during post-IS states. The first SEF has τ_{eff} = 56 sec and β = 0.87. These values are quite comparable with the post-NBS case. It suggests that there is a similar mechanism with the post-NBS during the recovery process. At the same time, the second SEF provides much larger value of τ_{eff} as 4507 sec, compared to any other case, and β = 0.11. In particular, this large value of τ_{eff} implies a persistent mechanism, so called PPC, which

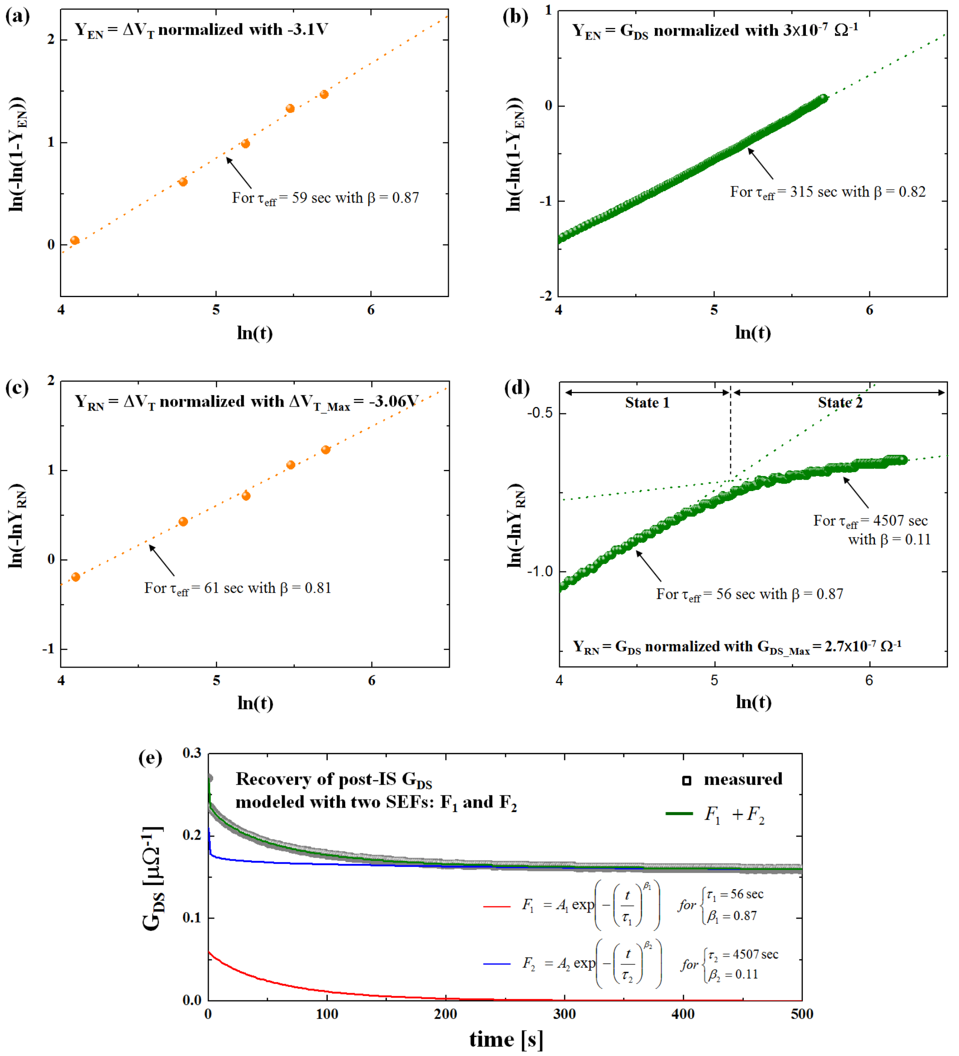

Fig.S1-1 Excitation behaviour modeled with SEF for (a) NBS and (b) IS, respectively. And recovery behaviours modeled with SEF for (c) NBS and (d) IS, respectively. (e) Recovery behaviour as a function of time for post-IS G_{DS} modeled with two SEFs. Here, A_1 and A_2 are constants and the sum of them equals to the value of G_{DS_Max}.

TABLE S1-I. SUMMARY OF PARAMETERS RETRIEVED FOR NBS AND IS CASES.

Stress Type	Excitation τ_{eff}	Excitation β		Recovery τ_{eff}	Recovery β
NBS	59 sec	0.87		61 sec	0.81
LS	315 sec	0.82	F_1	56 sec	0.87
			F_2	4507 sec	0.11

needs a much longer time to finish recovery, suggesting a contribution of oxygen defect contribution. The extracted values of τ_{eff} and β for each case are summarized in TABLE S1-I.

S2. Activation Energy Distributions with ILT

With the values of τ_{eff} and β, we can deduce an activation energy distribution. The first step is to get the frequency domain function, f(S), from the inverse Laplace transform (ILT) of F(t), as defined as follows,

$$L^{-1}\{F(t)\} \equiv f(S) = \frac{1}{2\pi j}\int_{-j\infty}^{j\infty} e^{-(t/\tau_{eff})^{\beta}} e^{St} dt, \tag{S2-1}$$

where S is a frequency. Replacing t/τ_{eff} with an imaginary parameter $j\omega$, Eq.(S2-1) can be rewritten with $dt = j\tau_{eff}\, d\omega$,

$$f(S) = \frac{\tau_{eff}}{2\pi}\int_{-\infty}^{\infty} e^{-(j\omega)^{\beta}} e^{j\omega\tau_{eff} S} d\omega. \tag{S2-2}$$

The solution of Eq.(S2-2) can be an analytic expression approximated with the saddle-point method, as given as,

$$f(S) \approx \frac{\tau_{eff}\beta^{1+\gamma/2}}{\sqrt{2\pi\beta(1-\beta)}\left(\tau_{eff} S\right)^{1+\gamma/2}} e^{-(1-\beta)\beta^{\gamma}/(\tau_{eff} S)^{\gamma}}, \tag{S2-3}$$

where γ is defined as $\beta/(1-\beta)$.

In order to get the activation energy distribution function from Eq.(S2-3), the frequency parameter, S, in Eq.(S2-3) needs to be converted into activation energy (E_A) using the Arrhenius relation,

$$S = \nu_{AE}\exp(-E_A/kT), \tag{S2-4}$$

where ν_{AE} is an attempt-to-escape frequency and kT the thermal energy. Note that ν_{AE} is different from a lattice vibration frequency which is about 10^{13} /sec.

Now, we have the activation energy distribution function, $f(E_A)$, based on Eqs.(S2-3) and (S2-4),

$$f(E_A) \approx \frac{\tau_{eff}\beta^{1+\gamma/2}}{\sqrt{2\pi\beta(1-\beta)}\left(\tau_{eff}\nu_{AE}e^{-E_A/kT}\right)^{1+\gamma/2}} e^{-(1-\beta)\beta^{\gamma}/\left(\tau_{eff}\nu_{AE}e^{-E_A/kT}\right)^{\gamma}}. \tag{S2-5}$$

S3. Determination of V_{AE}

In Eq.(S2-5), the main unknown is ν_{AE}. To retrieve the value of ν_{AE}, excitation state experiments under IS are performed for different temperatures, and each measured excitation plot is converted into an energy spectral function $F(E_A)$ rewritten based on 1-F(t) with Eqs.(S1-1) & (S2-4). So, $F(E_A)$ is represented as follows[19],

$$F(E_A) = 1 - \exp\left(-\left(\nu_{AE}\tau_{eff}\right)^{-\beta}\exp\left(\beta E_A/kT\right)\right). \tag{S3-1}$$

The $F(E_A)$ for each excitation case measured with different temperatures, e.g. 300K ~ 400K, is plotted as a function of E_A, for a different value of ν_{AE}, as shown in Fig.S3-1. The extracted values of τ_{eff} and β for each case are summarized in TABLE S3-I. It is found that all the plots for different temperatures, e.g. 300K ~ 400K, are well overlapped with each other when $\nu_{AE} = 10^7$ /sec. This suggests that the examined device has ~ 10^7 /sec as its attempt-to-escape frequency, which is consistent with the value reported in Ref: A. J. Flewitt and M. J. Powell, *J. Appl. Phys.* 115, 134501 (2014).

TABLE S3-I. SUMMARY OF PARAMETERS RETRIEVED WITH A STRETCHED EXPONENTIAL FUNCTION FOR DIFFERENT TEMPERATURES.

Parameters	Temperatures [K]				
	300	325	350	375	400
τ_{eff} [sec]	315	59	14	5	2
β	0.82	0.81	0.82	0.83	0.84

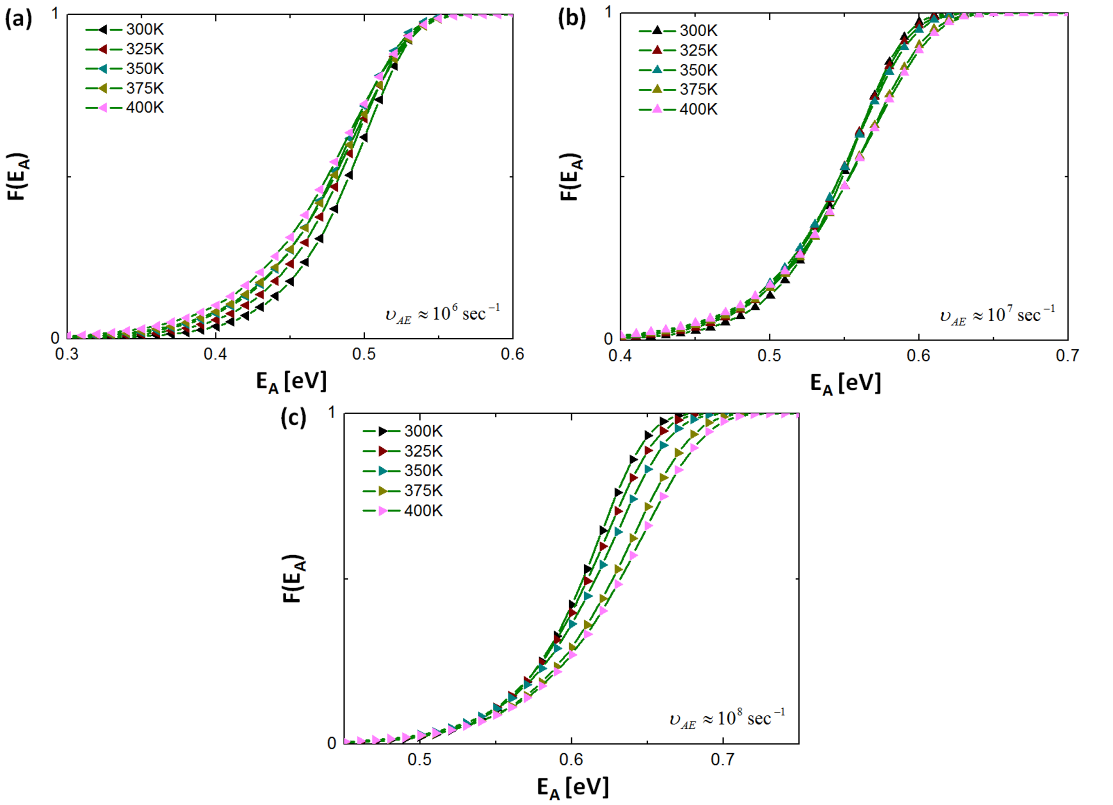

Fig.S3-1 Energy spectral function $F(E_A)$ for 5 different temperatures (i.e. 300K ~ 400K, step=25K) for three different values of ν_{AE}: (a) 10^6, (b) 10^7, and (c) 10^8 sec^{-1}, respectively.
